# Looking before you leap: A theory of motivated control of action

**DOI:** 10.1016/j.cognition.2009.03.006

**Published:** 2009-07

**Authors:** Elizabeth B. Liddle, Gaia Scerif, Christopher P. Hollis, Martin J. Batty, Madeleine J. Groom, Mario Liotti, Peter F. Liddle

**Affiliations:** a University of Nottingham, School of Community Health Sciences, Division of Psychiatry, E Floor, South Block, Queen’s Medical Centre, Derby Road, Nottingham NG7 2UH, UK; b Department of Experimental Psychology, University of Oxford, South Parks Road, Oxford, OX1 3UD, UK; c Department of Psychology, Simon Fraser University, RCB 5246, 8888 University Drive, Burnaby BC, Canada V5A 1S6

**Keywords:** Inhibition, Motivation, Stop Signal Reaction Time, Restraint, Control of action

## Abstract

The acquisition of volitional control depends, in part, on developing the ability to countermand a planned action. Many tasks have been used to tap the efficiency of this process, but few studies have investigated how it may be modulated by participants’ motivation. Multiple mechanisms may be involved in the deliberate exercise of caution when incentives are provided. For example, control may involve modulation of the efficiency of the countermanding process, and/or inhibitory modulation of the impulse to go. One of the most commonly used paradigms to assess control of action is the Stop Signal Task, in which a primary Go stimulus is occasionally followed by a countermanding Stop signal, allowing a *Stop Signal Reaction Time* (SSRT) to be inferred as the outcome of a “horse race” between the go and countermanding processes. Here, we present a computational model in which high task motivation modulates proactive pre-stimulus inhibition of the go response. This allows responses to be calibrated so as to fall within a time-window that maximizes the probability of success, regardless of trial type, but does not decrease the observed SSRT. We report empirical support for the model from a sample of typically developing children, and discuss the broader implications for operationalizing measures of volitional control.

## 1 Introduction

We often warn children to “be careful!” Impulsive action leads to accidents. And yet for many tasks, speed is also important. To cross a busy road we need to prepare to walk briskly, yet restrain the impulse to go until we have checked that the road is clear. Learning to control impulsive action is therefore an important aspect of development, and age-inappropriate levels of impulsivity are a clinically relevant symptom in developmental disorders such as Attention Deficit Hyperactivity Disorder (ADHD, American Psychiatric Association, 1994). Multiple models and empirical findings have brought into debate the question as to precisely how the control of action is implemented, both at the cognitive (e.g. Baddeley & DellaSala, 1996; Shallice, 1988), and at the neural level (e.g. Duncan, 2001; Goldman-Rakic, 1996; Miller & Cohen, 2001; Passingham, 1993; Stuss & Alexander, 2007).

In an influential paper, Logan and Cowan (1984) presented a theory of the control of action in the form of a *horse race*. Two independent processes were posited: an ongoing *go* process and a countermanding *stopping* process, each taking a variable time to complete. The “horse race” for action control is thus between an ongoing “go process horse” with a head start, and a faster “stop process horse” trying to catch up. If it succeeds in catching up before a ballistic *point of no return*, the response is successfully countermanded. If not, the “go process horse” wins, and the countermanding process fails. The relative values of two reaction times (RTs) are thus critical to the result of the race: the overt RT to the stimulus to act, and the covert RT to a signal to stop. This covert RT is widely referred to as the Stop Signal Reaction Time (SSRT).

Many subsequent studies have assessed limits and boundary conditions of the original model as an account of controlled action (e.g. Band, Ridderinkhof, & van der Molen, 2003; Boucher, Palmeri, Logan, & Schall, 2007; van den Wildenberg, van der Molen, & Logan, 2002). Further studies also demonstrated both overlap and differences across paradigms designed to investigate aspects of cognitive control, such as dual tasks and tasks requiring response countermanding, as well as tasks requiring control of competing responses (e.g. Kornblum, Hasbroucq, & Osman, 1990; Kramer, Humphrey, Larish, Logan, & Strayer, 1994; Logan & Burkell, 1986; Ridderinkhof, Band, & Logan, 1999). Converging cognitive and neural evidence suggests that control tasks requiring participants to inhibit certain responses or to countermand an initiated response rely on similar control mechanisms and underlying neural circuits. (For a recent review, see Aron et al., 2007.)

### 1.1 Inhibitory control of action

An experimental paradigm originally stimulating the development of the horse-race model and now routinely used for investigating the control of action is the *Stop Signal Task* (Lappin & Eriksen, 1966; Logan, 1983; Logan & Cowan, 1984), in which an imperative *Go* stimulus is presented, and on a minority of trials, a countermanding *Stop* signal is presented after a variable *Stop Signal Delay* (SSD). From the SSD value below which a response can be successfully inhibited, the time that elapses between the presentation of the Stop Signal and a successful inhibition of the response – the covert SSRT – can be inferred. The SSRT can be computed by various methods (Band, van der Molen, & Logan, 2003; Colonius, 1990; De Jong, Coles, Logan, & Gratton, 1990; Logan, 1981; Logan & Cowan, 1984) on the assumption that successful Stop trials are those in which, had those trials been Go trials, the reaction times (RTs) would have come from the slower tail of the population of RTs, whereas the RTs recorded on unsuccessful Stop trials are drawn from the faster tail of the distribution. This assumption is supported by the observation that the mean RTs recorded on failed Stop trials are almost invariably faster than the mean RTs recorded on Go trials (Band, Ridderinkhof, et al., 2003; Logan & Cowan, 1984). The SSRT can therefore be computed by taking the Go RT at the percentile corresponding to the proportion of unsuccessfully inhibited Stop trials and subtracting the mean SSD.

Although the horse-race model of control of action assumes independence of the go and stop processes, it is clear that if inhibition of the go process is to be achieved, the countermanding stopping process must interact with the ongoing go process at some point. Boucher et al. (2007) proposed a modification to the independent horse-race model based on processes known to operate during the generation and inhibition of saccades. Their purpose was to reconcile behavioural data consistent with an independent horse-race with known processes by which fixation and movement-related neurons interact to trigger or countermand a saccade. In their *interactive horse-race model*, the encoding of the countermanding signal is followed by rising activation in neurons governing the stopping process, which in turn inhibits the rising activation in neurons governing the go process. If the rise in go process activation is arrested before an irretrievable ballistic process is triggered, the saccade is successfully countermanded. The model was tested using parameters obtained from single cell recordings from monkeys engaged in the task. The authors concluded that the time that elapses between the point at which activation in the stopping process neurons starts to rise and the point at which the rising activation in the go process neurons is arrested is extremely short, and is dwarfed by the preceding period in which the Stop stimulus is encoded.

The duration of the SSRT – the time between the Stop stimulus onset and the inferred cancellation time of the go process – thus consists of three elements: a substantial period during which the Stop stimulus is encoded and which proceeds independently of go-related processes; a very short period during which rising activation in stop process neurons interacts with the Go-related process; and the duration of the independent ballistic process that would have occurred had the participant failed to stop.

However, if, as Boucher et al. (2007) suggest, the SSRT is composed largely of stimulus encoding and ballistic processes, even this modified interactive horse-race model of response inhibition is limited as a model of control per se, as the only free parameter remains the duration of the stimulus encoding process, which may or may not be under volitional control. Indeed, Logan and Cowan (1984) originally suggested that more sophisticated control mechanisms are likely to be relevant to the skilled control of action. For example, in many tasks, inhibitory and activation processes must be finely balanced to achieve a trade-off between speed and accuracy that is appropriate to current goals.

### 1.2 Motivation and control of action

Novel insights on cognitive control derive from the integration of the literature on response inhibition with that on motivation, reward processing and decision making. Learning models based on reward evaluation predict that motivational incentives should affect the efficiency of inhibition across a variety of cognitive control tasks, to the extent that these processes are under volitional control. Indeed, empirical findings and computational models suggest that cognitive control is strongly influenced by changes in perceived value, and that such value computations are extremely complex and multi-factorial (Montague, King-Casas, & Cohen, 2006; Schultz, Dayan, & Montague, 1997). If so, earlier accounts of the skilled control of action need to be modified to reflect the role of the valuation of likely outcomes in modulating the balance between inhibition and activation.

Evidence for such balance and its effects on multiple control parameters comes from both behavioural and electrophysiological findings. In Lappin and Eriksen’s (1966) original experiment, participants were asked to try to maintain their proportion of correctly inhibited responses at around 75%, while responding as rapidly as possible to the Go stimulus. Stop Signal Delay varied from block to block. The authors noted that to maintain the probability of inhibition at 75% in each block, participants had to lengthen their mean RTs by a value close to the SSD for that block, and observed that their results raised questions as to the processes governing voluntary control of RT. This question was later addressed by Ollman (1973), who postulated that participants may establish “deadlines”, postponing their responses so as to fall near the deadline, thus maximizing their chances of inhibiting their response should a countermanding signal be presented.

Evidence for voluntary shifting of the balance between speed (minimizing errors of omission) and caution (minimizing errors of commission) is also provided by a study by van den Wildenberg et al. (2002). They investigated the effect of reduced response readiness on RT during a Stop Signal Task with a two-forced-choice primary task, by inserting blocks of trials in which participants, in addition to inhibiting their responses following a Stop signal, had also to be wary of *No Go* trials. The authors observed longer mean Go RTs during these blocks, accompanied by a reduced probability of responding on Stop trials, suggesting a strategic shift of their participants’ priority from speed to caution.

Band, Ridderinkhof, et al. (2003) investigated speed-accuracy trade-off using a cued, two-forced-choice *Go/No-Go* task to compare behavioural performance and Event Related Potentials (ERPs) in two groups of participants, each of which undertook the task under one of two instructional conditions. In a *speed* condition, participants were instructed to respond as fast as possible, while in a *balance* condition, accuracy as well as speed was emphasized. The cue preceded the imperative stimulus by 1500 ms, allowing for response preparation to begin, and indicated whether a left hand or a right hand response should be prepared. In 20% of trials, the colour of the imperative stimulus indicated that the response should be withheld.

The authors found smaller amplitudes for the Contingent Negative Variation and Lateralized Readiness Potential (both preparatory ERPs) in the interval between cue and imperative stimulus, and slower Go RTs in the balance condition than in the speed condition. This finding suggests that when participants were more strongly motivated to be cautious, some kind of proactive inhibitory restraining process was engaged well before the presentation of the imperative stimulus, slowing Go reaction times, and increasing the probability of a successfully inhibited response in the event of a No-go trial. Alternatively, where speed was the primary motivator, Go processes may have been potentiated. Thus, while the encoding of the go and stop stimuli may be largely independent (in accord with the horse-race model), optimal performance on the task may require fine calibration of the balance between activation and inhibition of the response preparation process according to the motivational values assigned to speed and accuracy respectively.

Action control tasks frequently employ protocols designed to discourage strategic slowing, for example, by imposing time limits, and/or by rendering the inhibition rate independent of strategic slowing by the use of tracking algorithms that maintain an inhibition rate of 50% (Band, van der Molen, et al., 2003; Logan, Schachar, & Tannock, 1997; Osman, Kornblum, & Meyer, 1986; Sylwan, 2004). However, as the study by Band, Ridderinkhof, et al., 2003 would seem to indicate, and as Lappin and Eriksen (1966), and later Ollman (1973), noted, strategic slowing may itself index an important aspect of inhibitory control of action, reflecting the effects of changing instructional contingencies on intrinsic motivation.

The existing literature on the role of extrinsic motivation in modulating performance on the Stop Signal Task is limited to a few studies (Huang-Pollock, Mikami, Pfiffner, & McBurnett, 2007; Konrad, Gauggel, Manz, & Scholl, 2000; Michel, Kerns, & Mateer, 2005; Oosterlaan & Sergeant, 1998; Scheres, Oosterlaan, & Sergeant, 2001; Slusarek, Velling, Bunk, & Eggers, 2001), and is ambiguous, but generally suggests that motivational incentives affect inhibitory control, for example, by bringing performance by children with ADHD up to the level of typically developing children (Slusarek et al., 2001).

There is a body of evidence, therefore, to suggest that motivational factors, both intrinsic and extrinsic, modulate the efficiency of inhibitory control of action. How might these influences on action control be implemented? Is a single inhibitory process sufficient to account for them?

### 1.3 Mechanisms of inhibitory control

Schachar et al. (2007) proposed that in addition to *cancellation* processes following a countermanding signal, *restraint* processes implicated in behaviour observed during action control may be worthy of separate study, and that differing, if overlapping, circuits might be implicated in each. In order to study these distinct aspects of inhibitory control, the authors asked whether either or both of these processes function atypically in clinically impulsive children.

The finding of longer SSRTs in children with ADHD is robust (Jennings, van der Molen, Pelham, Debski, & Hoza, 1997; Logan et al., 1997; Nigg, 1999; Schachar, Tannock, & Logan, 1993), and has been interpreted as evidence for deficits in inhibitory control. Schachar et al. (2007) sought to tease apart the effects of restraint and cancellation on the observed SSRTs, and to ascertain whether children with ADHD would show deficits in both. The authors used two versions of the Stop Signal Task, in both of which the primary task was a visual two-forced-choice reaction task, and the Stop signal was an auditory tone. In a *cancellation* version, the Stop signal was presented after a variable SSD, computed using a tracking algorithm, while in a *restraint* version, the Stop Signal Delay was zero, rendering it comparable to a Go/No-Go task. The authors reasoned that in the cancellation version, the Stop signal would interrupt an already ongoing response process, whereas in the restraint version, children would delay the initiation of their response to the primary task stimulus until they had determined whether or not it was a Stop trial. The authors therefore proposed that the SSRTs in each version would index the efficiency of cancellation and restraint processes respectively.

The authors found that in both typically developing children and those with ADHD, SSRTs were longer in the restraint version than in the cancellation version, which they interpreted as evidence in support of their hypothesis that two separate processes might be involved, and concluded that children with ADHD had deficits in both. However, it is not self-evident that SSRT in the two versions accurately indexes both of these processes. In horse-race terminology, strategic restraint should delay the “go horse” rather than increase the speed of the “stop horse”, the cancellation process. If the participant strategically delays his/her responses, the percentage of failed responses on Stop trials will tend to decrease, thereby lowering the rank of the Go RT used to compute the SSRT, and leaving SSRT itself unchanged. Thus, the authors’ interpretation of their findings of longer SSRT in the restraint version as evidence for a separate restraint process may not be justified.

It remains unclear, therefore, which of multiple possible parameters modulating performance, including SSRT, may be under volitional control, bringing into question whether or not the longer SSRTs observed in participants with ADHD reflect impairments implicated in clinical impulsivity. One alternative interpretation is that the stimulus-encoding process in participants with ADHD is simply more time-consuming than in control participants, and may be irreducible beyond a certain working memory limit, as suggested in a review by Lijffijt, Kenemans, Verbaten, and van Engeland (2005), and which would not necessarily result in the clinically impulsive behaviour that is a symptom of the condition. In contrast, if the duration of the go preparation process is modulated by proactive partial engagement of restraint processes in anticipation of a signal to stop, as suggested by some of the evidence presented above, then deficits in volitional control of such strategic restraint processes may prove more relevant to clinical impulsivity than slow encoding of countermanding signals. However, such a deficit would not be indexed by the SSRT.

It is therefore pertinent to revisit the question originally posed by Lappin and Eriksen (1966) in their original paper regarding the processes governing voluntary control of the impulse to respond. What might participants actually be doing when they try to inhibit their response to a stimulus, and, by extension, what are those who exhibit apparently impulsive behaviour failing to do in such tasks? This paper poses the question as to whether, in a task in which the response to a Go stimulus may be randomly countermanded, increased motivation to avoid errors of commission results in shorter SSRTs, or, alternatively, whether it results in greater proactive restraint on the go preparation processes on all trials, and thus an increased probability of a successful inhibition should the trial prove to require it. Three alternative hypotheses as to how inhibition processes might be modulated by motivation are presented, and predictions flowing from these models are tested against data from a sample of school age children who undertook a version of the Stop Signal Task under three different motivational conditions.

### 1.4 Modelling motivated control of action

Following Logan and Cowan (1984), we assume that the response to a Go stimulus can be divided into two portions: an early portion during which the response can be inhibited; and a later ballistic portion that follows a point of no return, beyond which inhibitory processes cannot revoke the response. Also, like Logan and Cowan (1984), we assume that if the stopping process is completed before this point of no return is reached, a successful inhibition will result (a *signal*–*inhibit* trial), but that if the point of no return is reached before the completion of the stopping process, an error of commission will be made (a *signal*–*respond* trial).

**Fig. 1:**
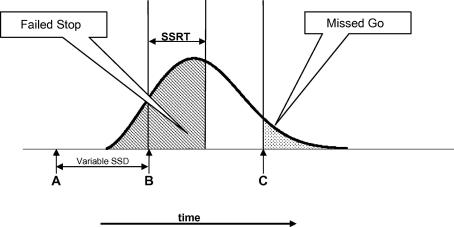
Diagrammatic representation of Stop Signal Task. Note: The primary stimulus is presented at Time A. On a minority of trials, a “Stop” signal is presented after a variable delay (“Stop Signal Delay” – SSD). The curve represents a histogram of response times. The model assumes that if the Stop Signal (B) is presented early enough that the SSRT will be completed during the period in which the response preparation is still within its “revocable” phase, the response will be successfully inhibited, but that if the response process has gone beyond the “point of no return”, the Stop process will fail. If, on a Go trial, the response falls beyond the time limit (C), the trial will be a “missed response” trial, i.e. a missed Go.

The Stop Signal Task is represented graphically in Fig. 1. At time zero, the primary task stimulus is presented (A). On a minority of trials, after a variable SSD, a Stop signal is presented (B). In this representation, a time limit for Go trials is also imposed (C). The curved line represents a distribution of durations of the revocable portion of a set of responses, and the double headed arrow represents that participant’s Stop Signal Reaction Time (SSRT). Any Stop trial in which the point of no return occurs earlier than the Stop signal (B) plus the SSRT will be a signal–respond trial. This population of signal–respond trials will therefore be drawn from the population of revocable response durations shown in the shaded area of the figure. Moreover, in a paradigm in which a time limit is imposed, any Go trial in which the point of no return is delayed so long that the resulting response time occurs later than the time limit (C) will result in a *missed-response* trial. This population of missed-response trials will be drawn from the distribution of revocable response durations shown in the dotted portion of the diagram. The successful Go trials (*hits*) will be drawn from the remainder of the distribution. How would motivation affect cognitive control within this framework?

If a participant is highly motivated to maximize the number of successful trials (trials that are either signal–inhibit or hits), three potential strategies are theoretically possible.

#### 1.4.1 Hypothesis 1

**Fig. 2:**
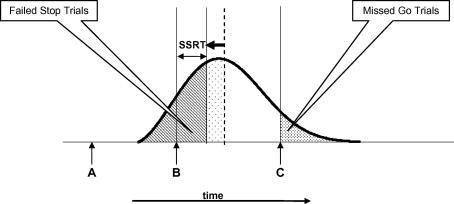
Hypothesis 1. *Note*: The participant reduces the duration of the SSRT, decreasing the proportion of failed Stop trials. There is no effect on mean RT of the Go trials, and the proportion of missed Go trials is unchanged.

Firstly, the participant might be able to reduce the duration of his/her SSRT. This would result in the pattern of performance illustrated diagrammatically in Fig. 2, in which a shorter SSRT leads to a smaller proportion of Stop trials that are signal–respond trials, and leaves unchanged the proportion of Go trials that are missed. This finding would be consistent with the focus on SSRT as a marker of action control in all existing theories of Stop Signal Task performance. If, as suggested by Boucher et al. (2007) the non-ballistic portion of the SSRT is largely taken up with encoding processes, then such a strategy would involve increasing the efficiency with which the Stop stimulus is encoded.

#### 1.4.2 Hypothesis 2

**Fig. 3:**
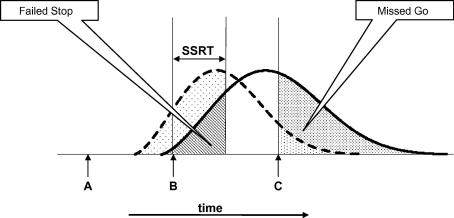
Hypothesis 2. *Note*: The participant globally delays all responses, increasing both mean and variance of RT distribution. The proportion of failed Stop trials decreases, but the proportion of missed Go trials increases. This strategy would result in a smaller population of Stop trials in which the response was unsuccessfully inhibited, but a larger population of Go trials in which the response was too late. It models the pattern of responses seen in Band et al.’s (2003)*balance* condition, in which participants’ responses were globally slower than that of those in the *speed* condition.

A second strategy that a participant might adopt would be to delay all responses by means of proactive, tonic, inhibition of the go process on all trials so that the point of no return is reached consistently later in the trial. This tonic restraint strategy is shown diagrammatically in Fig. 3, in which the dashed curved line represents the initial population of response durations (revocable portion), and the solid curve represents a population of durations that have been extended by engagement of a restraint process on all trials. Of note, this second strategy would leave the SSRT unaffected, but increase the proportion of missed-response trials, provided that a time limit is imposed. The adoption of this strategy would be consistent with the findings of Band, Ridderinkhof, et al. (2003) of a global increase in RT when participants were asked to balance speed against accuracy.

#### 1.4.3 Hypothesis 3

**Fig. 4:**
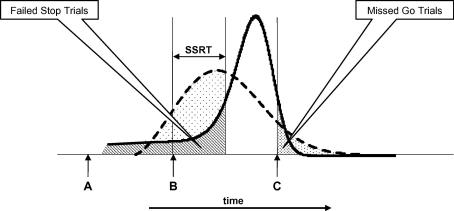
Hypothesis 3. *Note*: Participants target their responses on the time window in which success is most probable. Failed Stop Trials are reduced at no cost in missed Go trials.

A third strategy would be for the participants to time their responses more accurately so as to increase the proportion of their responses falling within the time window at which the probability of success is maximized. This strategy is shown diagrammatically in Fig. 4. The dashed curve again represents the initial population of response durations (revocable portion), and the solid curve a population of response durations in which a higher proportion fall within the time window that maximizes the chances of success – slow enough to give a fair chance of successful inhibition should a Stop signal be presented, but fast enough to be completed within the time limit should the trial turn out to be a Go. As with hypothesis 2, proactive restraint is postulated to be applied to go processes on all trials, but, unlike hypothesis 2, the restraint process is postulated to be one that is precisely calibrated trial by trial in order to target the optimal time window, rather than simply a tonic inhibitory set that globally increases the duration of the go processes on every trial. If this strategy were to be adopted, an increase in inhibition rate would be achieved without the cost of an increased miss rate, differentiating it from Hypothesis 2.

In summary, Hypothesis 1 predicts that motivation will affect SSRT by increasing the speed at which a signal to stop is encoded. In contrast, both Hypotheses 2 and 3 postulate that participants are able to restrain the go process itself, exercising voluntary control over the duration of the go process. However, whereas Hypothesis 2 postulates a global strategic slowing of all responses, at the cost of increased missed Go trials, Hypothesis 3 proposes that this control is precisely calibrated, trial by trial, in such a way as to maximize the probability of success if a countermanding signal is presented, while simultaneously minimizing the probability of missing a go stimulus.

The prediction made by Hypothesis 1 can be operationalized simply as a decrease in SSRT when the incentive to inhibit is increased. The prediction made by Hypothesis 2 can be operationalised as an increase in mean GoRT in response to greater incentive to inhibit. However, in order to operationalise the predictions made by Hypothesis 3, it was necessary to investigate what observable parameters might be modified by an optimization of the RT distribution in response to increased incentive to inhibit. In order to do this, firstly, a simple Bayesian statistical model was developed to capture the optimization parameters. We then used simulations to test how these might be modified by motivational incentives, and achieved by modulation of control parameters. Finally we operationalized the differential predictions of Hypotheses 1–3 using the findings from our model simulations.

**Fig. 5:**
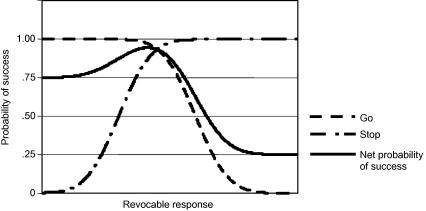
Bayesian model. *Note*: Time is represented along the horizontal axis, and each point along that axis represents the duration of the revocable portion of the RT. The vertical axis represents the probability of success. The dashed line represents the probability of success on Go trials for any given revocable portion of the response. A response with a short revocable portion will result in a response time with a high probability of being within the time limit. As the duration of the revocable portion is lengthened, the probability that the total response will be completed within the time limit rapidly reduces to zero. The slope of the line is a function of the variance in the ballistic portion: the smaller the variance in the ballistic portion, the steeper the slope down to zero will be. As the length of the revocable portion of the Go process increases (*X* axis), the probability of success on Go trials (timely response) decreases (dashed line) and the probability of success (inhibited response) on Stop trials increases (dot-dashed line). The solid line represents the net probability of success on each trial, given a 25% probability of a Stop signal on each trial.

Our Bayesian model is shown in Fig. 5. The dot-dashed line represents the probability of success on Stop trials for any given revocable response duration for Stop trials. The longer the duration of the revocable portion, the more likely it will be that the Stop Signal will have occurred before the “point of no return” is reached, allowing the response to be successfully inhibited. The upward slope of the line is therefore a function of the distribution of SSDs specified by the experimenter. The solid line in the figure is the summed probability of overall success, given the probability of Go and Stop trials respectively. For any given revocable response duration, the probability of success for any trial is given by:

(1) p(success)=p(success|Go)∗p(Go)+p(success|Stop)∗p(Stop)

where *p*(s*uccess*) is the probability of success, *p*(*Go*) is the probability of a Go trial and *p*(*Stop*) is the probability of a Stop trial.

It can be observed from Fig. 5 that the revocable response duration with the maximum probability of overall success (solid line maximum) occurs near the intersection between the probability of success, given a Go trial, and the probability of success, given a Stop trial. However, because of the preponderance of Go trials (the figure illustrates a 75% probability of Go trials), the rise that precedes the optimum duration is less steep than the drop-off in probability of success after the optimum point is reached, resulting in an asymmetric function with a higher shoulder on the faster (left) side of the peak than on the slower side.

This curve thus represents a fitness function: responses that are timed so that their revocable portions terminate under the higher portions of the curve are “fitter” – more likely to be successful – than responses in which those portions terminate under lower portions of the curve. Thus, the peak of the curve represents a “sweet spot” in which the overall probability of success is maximized, regardless of trial type. Note that, because of the asymmetry of the curve, a strategy to calibrate the degree of restraint in such a way that optimizes the simple probability of success would result in a distribution of RTs with a peak at the “sweet spot”, but with a greater prevalence of fast responses to the left of the peak than of slow responses to the right.

Such a distribution was modelled for the purposes of producing Fig. 1 simply by raising the values for the probability of success given by Eq. (1) to a power. Raising the values to a power less than 1 flattens the distribution, representing behaviour with reduced accuracy, while a power greater than 1 increases the frequency of responses at the optimum, and thus represents more accurate performance. However, in practice, such behaviour must be learned by probing the fitness landscape with actual or hypothetical responses, and receiving reinforcement signals (Montague et al., 2006). For example, a learning algorithm in which the responses associated with success were more likely to be repeated, while those associated with failure were suppressed, would enable the “fitness landscape” to be explored by trial and error, and the higher areas to become more “populated” with response times than the lower areas. Furthermore, any change in the relative value of the reinforcement given to Stop and Go trials will alter the fitness landscape itself, and thus the learned distribution of RTs. For example, if successful performance on Stop trials is valued more highly than that on Go trials, the peak corresponding to maximal “fitness” will tend to move rightwards, as longer responses net a greater yield. One consequence of such a change in the shape of the distribution will be that the portion of the distribution corresponding to signal–respond trials will be in a lengthening negative tail. In other words, the model makes the unique prediction that when motivational incentives to succeed on both trial types are finely balanced, the shape of the RT distribution will change to be more negatively skewed than when motivation to avoid an error of commission is reduced. One consequence of this change in RT distribution will be that the difference between mean RTs on Go trials and those on signal–respond trials will tend to increase as the bulk of RTs moves rightwards. This suggests that the difference between Go RTs and Stop (signal–respond) RTs may be a useful index of such a change in the distribution.

### 1.5 Reinforcement learning model

Reinforcement-learning models are based on the assumption that organisms learn to achieve goals by means of reinforcement signals received following a given action (Montague et al., 2006). Learning will occur if an action that brings the organism closer to its goal is more likely to be selected on future occasions (or, conversely, if an action that counters the achievement of that goal is less likely to be selected). Thus, the reinforcement signal can be seen as modulating the value of the successful action, and this value can be considered as a re-weighting of that action with regard to future decision-making: actions with increased value have an increased probability of being selected on future occasions, and vice versa.

We therefore programmed an exploratory learning model in Matlab 7.3 (The MathWorks, 2006). The paradigm emulated was a Stop Signal Task with a tracking algorithm similar to that used by Logan et al. (1997), in which the SSD was incremented after a successful inhibition, and decremented after a failed inhibition, with a constant time limit (*T*) for each trial. Response times to the primary stimulus were modelled as the sum of three components: a fixed encoding time (*E*) (an arbitrary constant); an adjustable delay (*D*); and a ballistic component (*B*) with a duration randomly drawn from a quasi-Gaussian distribution (a Weibull distribution with a shape parameter of 3.6). The sum of the constant *E* plus the variable *D* was taken to represent the revocable portion of the go process, with the *D* component representing the lengthening effect on the go process of a restraining inhibitory process that was subject to trial-by-error learning. The underlying SSRT, representing the duration of the stop process, was assumed to consist of an irreducible encoding duration. followed by an instantaneous cancellation of the go process, and initially modelled as an arbitrary constant (*S*) (selected to be shorter than the go signal encoding constant *E*, in line with experimental data indicating that SSRT tends to be shorter than RT); in later implementations of the model the SSRT was modelled as a distribution. (Note that subsequently the observed mean SSRT over a sequence of trials was computed as the RT at the percentile corresponding to failed inhibition rate, minus the mean SSD for that sequence of trials, and did not necessarily correspond precisely to the modelled underlying SSRT.)

A random 25% of “trials” were deemed to be Stop trials, and the rest were deemed to be Go trials. On Stop trials, a Stop signal was deemed to have been presented after the selected SSD. If the revocable portion of the go process (*E* *+* *D*) on any trial was greater than the stop signal delay plus the duration of the stopping process (*SSD* + *S*) for that trial, a signal–inhibit was deemed to have occurred, as the cancellation process was deemed to have been completed before the point of no return (*E* *+* *D*), and the SSD was incremented for the next Stop trial. Conversely, if the revocable portion of the go process (*E* *+* *D*) was less than the signal delay plus the duration of the stopping process (*SSD* + *S*) for that trial, a signal–respond trial was deemed to have occurred, and the SSD was decremented for the next Stop trial. Similarly, on any Go trial, if the total go process duration, including the ballistic portion (*E* *+* *D* *+* *B*) was less than the time limit (*T*), the trial was deemed to have been a hit, while if the total go process duration was greater than *T*, the trial was deemed to have been a missed-response trial.

On each trial, the duration *D* was randomly retrieved from a distribution of *D* durations, represented as a vector of values and multiplied by a random “mutation” parameter. The initial *D* distribution was generated by a Weibull function with a shape parameter of 2, which gives a distribution with a positive skew, typical of a distribution of reaction times in a simple reaction time paradigm. Thereafter, following a successful trial, *V_trial__type_* copies of the *D* duration selected for that trial were appended to end of the vector representing the *D* distribution, and the first *V_trial_* *_type_* values were deleted, where *V_trial__type_* was an adjustable non-negative integer representing the “value” accorded to success on that trial type. Thus, the *D* distribution from which each trial *D* duration was drawn became gradually enriched by recently successful values of *D* and the probability of a recently successful *D* duration being selected on future trials increased. The “mutation” parameter was drawn from a Weibull distribution with mode of 1 and a shape parameter of 2, and raised to a fractional power.

The learning algorithm is thus based on a similar principle to that of Logan (1992) “instance theory” of learning, and is, essentially, an evolutionary algorithm by which the fitness landscape is explored. Moreover, the “mutation” parameter ensured that the model was able to respond to changes in the fitness landscape (e.g. changes in the valuation of each trial type) just as a minimum mutation rate is required to prevent populations of organisms becoming stranded on local maxima. The shape of the distribution of the “mutation” parameter was chosen to match the shape of the initial RT distribution, raised to a fractional power to reduce its variance. These properties meant that the “learned” distribution is repeatedly convolved with a distribution with the same shape as the initial distribution, so that in the absence of any “incentive” to “remember” successful *D* values (when the value of *V_trial__type_* is low or zero, and few or no successful values are replicated in the *D* distribution), the distribution of *D* tends to revert to the initial distribution, thus emulating the extinction of the learned behaviour in the absence of reinforcement.

In order to assess the impact of incentive to succeed on the distribution of RTs, we manipulated the balance between the “value” of Stop trials and the “value” of Go trials. Because in a standard Stop Signal task the Stop trials are outnumbered by the Go trials, in order to model a condition in which the net value of the two trial types were finely balanced, we accorded a higher “value” to *D* durations resulting in a successful Stop trial than to delay values resulting in a successful Go trial. We contrasted the behaviour of the model under this “high motivation” condition with a “low motivation” condition in which only delays resulting in a successful Go trial had a non-zero “value”, and there was no counter-incentive to “avoid” an erroneous response on a stop trial.

The model was tested by running it under these two alternating motivational conditions for a range of values of the free parameters. Runs consisted of 50 consecutive “sessions”, each session consisting of 50 “blocks”, each with 200 “trials”. In the initial two “sessions”, each “value” of both trial types was set to zero, enabling RT distributions to be collected before “learning” had begun; thereafter, “high motivation” sessions were alternated with “low motivation” sessions. No changes were made to any other parameter, and the final RT distribution from each block was carried forward to the next session. “Observed” SSRTs were computed for each block as the Go RT at the percentile corresponding to the failed inhibition rate (Lappin & Eriksen, 1966) minus the mean SSD for that block. Miss rate, mean Go RTs, mean failed Stop RTs, and Go-Stop RT differences (Mean Go RT minus mean failed Stop RT) were also computed for each block.

We report here results from a run in which, following the first two sessions in which no value was accorded to successes on either trial type, the even-numbered “sessions” were “high motivation” sessions in which the value accorded to successful Stop trials was set to 10, and the value of *V_trial__type_* accorded to successful Go trials set to 2. The odd-numbered “sessions” were “low motivation” sessions, in which the value of *V_trial__type_* for Stop trials was set to 0 and the value of *V_trial_* *_type_* for Go trials to 1. The setting of a minimal non-zero value for successful Go trials in the “low motivation” condition proved necessary to ensure that the mean RT did not rise unconstrainedly, and that the miss-rate was maintained at below 50%, as observed in actual participants. The mean value of the initial distribution of RTs was set at 400 units, consisting of 350 units of “encoding time” (*E*), a mean of 40 units of “delay” time (*D*) and a mean of 10 units of “ballistic” time (*B*). In all runs of the model using these parameters, inhibition rate converged rapidly to 50% (owing to the tracking algorithm) during the first “session” and miss rate stabilized at 33%, by the end of the 6th “session”.

**Fig. 6:**
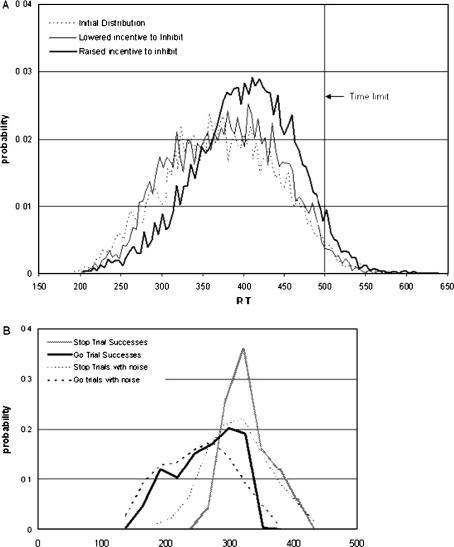
Sample output from the inhibition learning model. *Note*: (A) represents probability density functions for the distributions of Go RTs at the start of the run (dotted line; left-hand vertical axis); during a later “high motivation” session (heavy solid line); and a later “low motivation” session (light solid line). The initial distribution is positively skewed, by design. The distribution during the “high motivation” condition has a sharper peak, the mode has shifted to a longer value, and it is negatively skewed. The distribution during the “low motivation” condition closely resembles the initial distribution, the distribution that was learned during the “high motivation” condition having been extinguished through lack of reinforcement of success on the Stop trials. (B) represents the probability density functions for the delay values (“*D*”), as both stored (solid lines) and retrieved (dotted lines). The grey solid and light dotted lines represents values of “*D*” resulting in successfully inhibited responses on Stop trials, and the black solid and heavy dotted lines represent values resulting in timely Go responses. The dotted lines represent values retrieved by the model, i.e. after multiplication by the “mutation” parameter. The “learned” distribution of RTs in (A) (heavy solid line) can be thought of as the sum of these two retrieved distributions, with the addition of variance contributed by the ballistic portion of the Go response, and the “encoding” constant, *E*.

Sample Go RT distributions from the model are plotted in Fig. 6A. The dotted line represents the distribution before any “learning” has occurred, resembling a typical RT distribution with a positive skew. The heavy solid line represents the distribution during the final “high motivation” session, when the model has “learned” an optimum RT distribution. The mode has shifted to a longer value, representing the “sweet spot” – the peak of the fitness landscape at which success is most probable – and the distribution is now negatively skewed. The lighter solid line represents the RT distribution from the final “low motivation” session; without any “incentive” to inhibit response on Stop trials to counterbalance the incentive to avoid a missed “Go” trial (i.e. without maintenance of a high probability of repeating a *D* duration that resulted in a successful Stop trial), the Go RT distribution has reverted to one resembling the initial distribution.

The reason for the characteristic shape of the Go RT distribution from the “high motivation” becomes apparent from examination of Fig. 6B. The “learned” distribution of successful delays when both trial types are valued can be thought of as the sum of two distributions: the distribution of delays that resulted in a correctly inhibited response on a Stop trial, and which will tend to have longer values of *D*; and the distribution of delays that resulted in a timely response to a Go trial, and which will tend to have shorter values. In Fig. 6B, these *D* distributions are plotted, both as stored after a successful trial and as retrieved for a subsequent trial. Extreme values have been systematically eliminated from both distributions, and the modes of the two distributions have approached each other, producing a peak in the combined distribution representing optimal values. The “population” of restraint values has thus “colonized” higher regions of the fitness function given by Eq. (1), changing the distribution from one with a longer positive tail to one with a longer negative tail, and consequently increasing the difference between the mean RT on failed Stop Trials and the mean RT on Go trials.

**Fig. 7:**
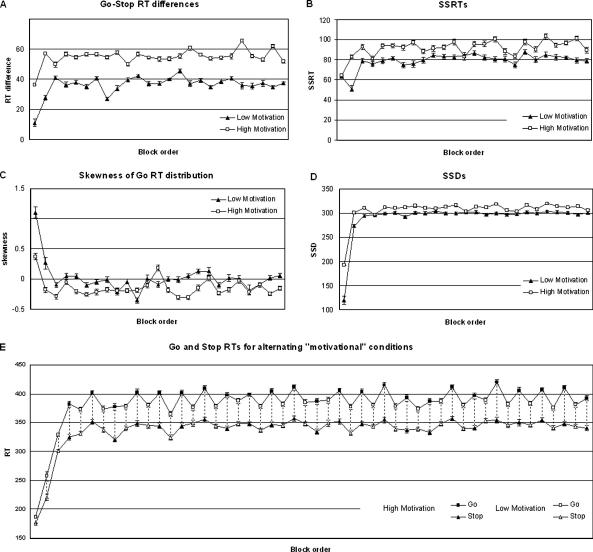
Typical model output. *Note*: Model output for a series of 50 “sessions”, each consisting of 50 “blocks” of 200 “trials”. In the first two sessions, the “value” accorded to each trial type was set to zero. Thereafter, “low motivation” sessions (value of Go trials = 1, value of Stop trials = 0) alternated with “high motivation” sessions (value of Go trials = 2, value of Stop trials = 10). “Low” motivation blocks are shown as open symbols, “high motivation” blocks are shown as filled symbols. Error bars represent the standard error. In plots (A–D), “high motivation” and “low motivation” sessions are shown as separate lines, in the order presented. Go-Stop RT differences were consistently greater on “high motivation” sessions, skewness was more negative, and SSD was lower, as predicted. Although the underlying SSRT was constant, by design, the observed SSRT was consistently longer on “high motivation” blocks, suggesting that a more negatively skewed Go RT distribution may result in a more overestimated SSRT. In plot (E), all 50 alternating sessions are represented in sequential order on the *X* axis, and both Go and failed Stop RTs are plotted for each, showing how the RT difference is consistently greater in the high motivation sessions, even though the mean failed Stop RT is sometimes shorter and sometimes longer.

The mean and standard errors for the Go-Stop RT differences, the SSRTs, the SSDs, the skewness parameters, and the Go and failed Stop RTs for each block within each session were computed for the same run, and are presented graphically in Fig. 7. Effect sizes (Cohen’s *d*) were computed, using an independent samples *t* test on the block means for those sessions after the model had stabilised (sessions 5–50). As predicted, the Go-Stop RT differences are consistently greater in the “high motivation” sessions than in “low motivation” sessions, reflecting the more negative skew of the Go RT distributions, the effect being apparent by the end of the first block of 200 trials, and remaining stable for the rest of the session. On this run the effect of motivational condition on the Go-Stop RT difference was 1.6. The skewness statistic has a large associated standard error; nonetheless the statistic tended to be more negative in the “high motivation” than in the “low motivation” sessions on all runs, although the effect size was smaller than for the Go-Stop RT difference (Cohen’s *d* = .35). The correlation between Go-Stop RT difference and the skewness statistic was also computed for these sessions, and for this run was significantly negative (*r* = −.17, *p* < .001, *N* = 2300), indicating that Go-Stop RT may be a good proxy measure for skewness in this task with a larger effect size for a given degree of variance. As expected, the SSD was consistently lower in the “high motivation” sessions (Cohen’s *d* = .6), reflecting a “poorer” inhibitory performance by the model when the value accorded to successful Stop trials was lowered.

In Fig. 7E, both Go and failed Stop RTs are plotted for each session. As would be expected, when the model “tries” to increase inhibition rate (although thwarted by the algorithm, which raises the SSD in response, as shown in Fig. 7D), the mean Go RTs tend to be longer than in the “low motivation” condition. However, to maintain a high hit rate, the peak of the distribution has to be kept under the time limit, pushing the whole distribution, including the portion consisting of short RTs, leftwards (faster). For this reason, the mean of the left hand portion of the distribution from which failed Stop RTs are drawn is often faster than in the “low motivation” condition.

Interestingly, although the SSRT was modelled as a constant, the observed SSRT was consistently longer than the modelled value (mean = 81, SD = 19 units, compared with a modelled value of 55 units) and consistently longer still in the “high motivation” blocks (mean = 93, SD = 19 units), suggesting that when RT distributions are more negatively (or less positively) skewed, the inferred SSRT is over-estimated. Cohen’s d for the effect of condition on the observed SSRT was .6. Moreover, the correlation coefficient between skewness and SSRT was significantly positive (*r* = .362, *p* < .001, *N* = 2300). It is worth noting, however, that the bias in the estimate of the SSRT induced by a change in the skewness of the Go RT distribution was in the opposite direction to that which would be observed in actual participants if they were able to reduce their RT in response to an increased incentive to inhibit their responses on Stop trials: When delays resulting in a successful Stop trial were more highly valued, although the underlying SSRT remained, by design, constant, the observed SSRT nonetheless increased.

Similar results to those presented here were obtained when the high motivation condition was presented on even numbered blocks, and also when the underlying SSRT was modelled with non-zero variance (values drawn randomly from a Weibull distribution with a shape parameter of 3.6, which gives an approximation to the normal distribution), although the effect of condition on SSRT was reduced in size.

### 1.6 Empirical validation

As modulation of control by motivational incentives has generally only been explored in the context of a comparison between children with and without impulsivity (Huang-Pollock et al., 2007; Konrad et al., 2000; Michel et al., 2005; Oosterlaan & Sergeant, 1998; Scheres et al., 2001; Slusarek et al., 2001), we proposed to test these models in a sample of typically developing children in their own right. Indeed, we believe action control processes should be first understood in individuals without impulsivity, in order to more clearly interpret performance differences by individuals with impulsivity. Of note, because of its childhood onset, impulsivity in ADHD is tested in children rather than adults, further motivating our choice of a sample of children for our current investigation.

Following from our modelling exercise, differential predictions flowing from our three hypotheses were operationalized as follows:

#### 1.6.1 Hypothesis 1

If participants were able to shorten their SSRTs in response to greater incentive to inhibit, we should see shorter mean SSRTs under more motivating conditions (Fig. 2).

#### 1.6.2 Hypothesis 2

If participants tended to strategically slow their responses on all trials, we should see a higher rate of missed “Go” trials under more motivating conditions, as well as globally longer RTs (Fig. 3). In addition, because, when RT increases, RT variability increases (Wagenmakers & Brown, 2007), under this hypothesis one might also expect to see an increase in the difference between mean Go and mean failed Stop RTs. However, if RTs were globally increased without any change in the shape of the RT distribution, any increase in Go RT or in the difference between Go and failed Stop RTs should be accompanied by an increase in the proportion of missed Go trials.

#### 1.6.3 Hypothesis 3

If participants timed their responses more precisely in order to maximize their chances of success on both types of trial, we should see an increase in the difference between mean RTs on Go trials and mean RTs on signal–respond trials under more motivating conditions. No effect would be expected on miss rates if this strategy was adopted. Thus, Hypothesis 3 would be supported if we observed such a pattern of mean response times in the absence of any increase in the rate of missed responses (Fig. 3).

## 2 Method

### 2.1 Participants

Thirty-three children (23 boys and 10 girls) with a mean age of 8 years 9 months (SD = 20 months) were recruited from local schools. Ethics approval was obtained from the Ethics Committee of the School of Psychology, University of Nottingham, and written informed consent was received from the primary care-giver of each child.

### 2.2 Procedure

A two forced-choice version of the Stop Signal Task was presented on lap-top PCs with 14” monitors, and a two button mouse (not a response box), was used for responses, easing the collection of data in school settings, at a small cost in precision. Two lap-tops were taken to schools and set up in a spare classroom, and two children at a time undertook the task. The task was programmed in E prime (Psychology Software Tools Inc.) and presented as a computer game. The experimental task was preceded by a short practice block of 12 Go trials, which was repeated until block accuracy reached 80%. Following this, three practice blocks of the Stop signal version of the task were presented introducing each motivational condition. At the conclusion of the practice, a time limit was computed for each child on the basis of their correct Go RTs (time limit = mean Go RT + 3 SD) and used in the experimental task.

The task was themed as a “space journey”. On the lap-top screen was a representation of a starry sky seen through a rectangular spaceship “porthole”. Each child was told that the task was to steer a space-ship to a distant planet, represented by a coloured disk 8 mm in diameter in the centre of the computer screen. Each “planet” was presented between a pair of grey square brackets (height = 20 mm; distance apart = 30 mm). Each child was told that they would need to steer round “gas clouds”, and that the grey brackets were “gas cloud detectors” mounted in the porthole glass. If the bracket on the left glowed red, they should press the left mouse button, and if the bracket on the right glowed red, they should press the right mouse button. If they pressed the wrong button, or were too slow, a “gas cloud” would appear on screen. The gas cloud image, if it appeared, remained on screen for 600 ms, during which time it masked the planet. Thus the red bracket served as the target stimulus, and remained illuminated until the time limit for response had elapsed. The gas-cloud stimulus itself provided negative feedback for erroneous or slow responses. The children were told to keep watching the “planet” between the two “gas cloud detectors”, which remained on screen across trials. The “planet” remained visible except when the “gas cloud” was presented, serving to help maintain fixation between the “gas cloud detectors”. Gaze direction was not systematically monitored, but two experimenters remained present at all times to ensure task compliance.

The child was then warned that sometimes a “pet alien” would “jump out” in front of the space-ship, and that if this happened, they were to try not to press the mouse buttons. The pet alien image thus served as the Stop signal, and took the form of a circular black and white image of a space alien (diameter = 25 mm), presented for 100 ms at centre screen, temporarily masking the fixation (“planet”) image, but leaving the Go stimulus (red bracket) visible. A tracking algorithm was used to compute the SSD for each trial: The initial SSD was set at 200 ms; after each successfully inhibited Stop trial, the SSD was incremented by 33 ms, and after each unsuccessfully inhibited Stop trial the SSD was decremented by 33 ms. The time interval between the offset of each Go stimulus and the onset of the next was randomly varied between 1200 and 2000 ms.

Three motivational conditions (*positive, negative,* and *neutral*) were indicated by three differently coloured “planets” and by a rectangular picture (500 x 400 mm) of an “inhabitant” of that planet displayed in the upper right hand corner of the screen. Children were told that on each of five “delivery trips” they would deliver various items to each of the three planets. Each “planet” was thus “visited” a total of five times. On one planet (positive), the inhabitants would pay 1 point each time the child missed a gas cloud (by responding correctly under the time limit), and 5 points each time they avoided hitting the pet alien. On another planet (negative), the inhabitants would deduct 1 point every time they hit a gas cloud (by being too slow, or responding incorrectly) and deduct 5 points every time they hit the pet alien. On a third planet (neutral) the inhabitants would neither pay nor deduct points. Within each of the five blocks (“delivery trips”), the order of the three conditions (“planets”) was randomized. Children were given 50 points to start with, and at the end of each visit to a planet, they were given a breakdown of their point score for that “visit”, as well as their overall running score. In addition, at the end of each visit, a short animation sequence unique to each planet was presented, in order to maintain motivation and to emphasize the difference between the conditions. Point totals were presented on an analogue scale consisting of a bright green vertical bar, and as a numeric value. Each “visit” to a planet consisted of 36 trials, giving 180 trials for each condition, of which 45 (25%) were Stop trials. At the end of each complete block (“delivery trip”), an additional short animation sequence was shown, together with information about how many “delivery trips” had been completed and remained.

### 2.3 Analysis

Mean inhibition rates were computed for each child for each condition by dividing the number of successfully inhibited Stop trials by the total number of Stop trials. One-sample *t* tests were used to ascertain whether the tracking algorithm had succeeded in maintaining mean inhibition rates for each condition at 50% across the sample. As accuracy rates on the primary task were high (close to the ceiling of 100%), the mean and standard deviation of the percentage scores would have been poor estimates of central tendency and dispersion, respectively. Accuracy rates were therefore first normalised with a *p* to *z* transform, the mean and confidence intervals computed, and the statistics then converted back to percentage values for purposes of presentation. SSRTs were computed for each child for each condition as follows: Incorrect Go trials were discarded, and the mean SSD for each condition was subtracted from the correct Go RT corresponding to the percentile representing the proportion of failed Stop trials for that condition. Thus, for a condition in which a child failed to inhibit on 48% of Stop trials, the mean SSD was subtracted from the correct Go RT at the 48^th^ percentile. Miss rates for each condition were computed by dividing the number of trials in which no response was recorded before the time limit by the total number of Go trials. Mean RTs for correct Go trials and mean RTs for Stop trials in which a response was made were also computed. An alpha level of .05 was used for all planned comparisons.

As the planned comparison of interest was between neutral and reinforced conditions, differences between the mean values of each dependent variable (SSRT; miss rate; Go-Stop RT difference) in neutral and reinforced conditions were computed, and checked for outlying values. One-way repeated-measures ANOVAs with three levels of motivational condition (neutral; positive and negative reinforcement) were then conducted on SSRTs and miss rates, and a two way repeated-measures ANOVA with the same three levels of condition, and two levels of trial type (Stop and Go) was conducted on the mean RTs. Planned comparisons were made between Go-Stop RT differences in the reinforced conditions (positive and negative) and the neutral condition, then between the two reinforced conditions.

### 2.4 Results

The children generally appeared to enjoy the experiment. The mean and 95% confidence limits of the accuracy rates for the primary Go task were as follows: mean = .95; lower cl = .86; upper cl = .98. Three children completed only four blocks due to time constraints, and the remainder completed all five blocks. One male participant with an outlying Go-Stop RT difference that was 3.9 standard deviations from the group mean was excluded from subsequent analyses, leaving 32 participants in the sample. All other difference values were within three standard deviations of the group mean. Means and standard deviations for the inhibition rates, miss rates, SSRTs, SSDs, Go RTs and RTs on failed Stop trials (Signal–Respond) RTs are given in Table 1.

One sample *t* tests indicated that the tracking algorithm had been successful in producing inhibition rates that were not significantly different from 50% for any condition, the largest variance for any condition being just over 5 percentage points.

### 2.5 Hypothesis 1

We had hypothesised that SSRTs would be shortened when incentive to succeed was greater. One-way ANOVAs indicated that there was no significant difference (*F* < 1) between SSRTs for each condition. The confidence limits for the mean change in SSRTs when an incentive to inhibit was provided were −8 ms (a shortening) to +15 ms (a lengthening). As the study had 87% power to detect a medium effect (Cohen’s *d* > 0.5) in the hypothesised direction, we can conclude that any shortening effect of an incentive to succeed on the SSRT was likely to have been small.

### 2.6 Hypothesis 2

We had hypothesised that participants would globally slow all responses when the incentive to succeed was greater, resulting in an increase in miss rates, and/or a global slowing of Go RTs. The one-way ANOVA conducted on the miss-rates indicated no significant difference in miss rates (*F* < 1) between the three motivational conditions. The confidence limits for the mean change in miss rates were −.03 (a decrease) and .01 (an increase). Again, as the study had 87% power to detect a medium effect in the hypothesised direction, we can conclude that any tendency for a greater incentive to succeed to result in an increase in missed Go trials was unlikely, or at best a small effect.

### 2.7 Hypothesis 3

**Fig. 8:**
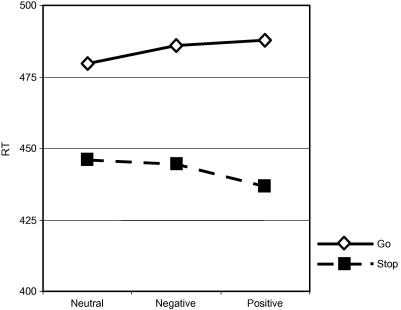
Reaction times by trial type and motivational condition. As anticipated, mean RTs (in milliseconds) for failed Stop trials are shorter than for Go trials. This difference is increased in the reinforced trials, consistent with the hypothesis that responses are targeted in a time-window that will optimize success.

A two-way ANOVA (trial-type by condition) conducted with mean correct Go RTs and mean failed Stop RTs, indicated a significant interaction between condition and trial, *F*(2, 32) = 7.23, *p* < .01, suggesting that condition had a significant effect on Go- Stop RT differences. Inspection of the means indicated, in support of Hypothesis 3, that RT differences were greater under conditions in which the incentive to succeed was greater (see Table 1). An expected main effect of trial type was also significant *F*(1, 31) = 100.19, *p* < .001, indicating that mean RTs on signal–respond trials were faster than mean RTs on hits, consistent with a horse-race model (Logan & Cowan, 1984). There was no significant main effect of condition (*F* < 1). The results from this two-way ANOVA are plotted in Fig. 8.

A planned comparison between the Go-Stop RT differences in the neutral condition with those in the two reinforced conditions indicated that the differences were significantly greater in the reinforced conditions, *F*(1, 31) = 11.92, *p* < .01 than in the neutral condition. The effect size (Cohen’s d) for the difference was 0.58, a medium effect size. Comparison between the differences in the two reinforced conditions indicated that the mean differences were greater in the positive than the negative condition, but this difference did not reach statistical significance, *F*(1, 31) = 3.70, *p* = .06 (See Table 1.)

## 3 Discussion

The finding of a significant effect of reinforcement condition on participant behaviour confirms that participants were responsive to motivational manipulation. Indeed, children frequently expressed strong opinions about the task, described the neutral condition as “boring”, and said that they liked the positive condition the best. This, together with the evidence that reinforcement condition was predictive of their Go-Stop RT differences suggests that reinforcement did influence behaviour. Nonetheless, no significant difference in mean SSRTs was found between conditions, nor was there any trend to significance. Our findings therefore do not support Hypothesis 1, namely that given increased incentive to inhibit, participants would shorten their SSRTs. However, it should be pointed out that our modelling exercise indicated that a more negative skew in the Go RT distribution results in a greater overestimate of the SSRT. Given the evidence in our study of an increased skew in Go RT distribution, as indexed by the observed increase in Go-Stop RT difference, it is possible that a real decrease in SSRT in response to increased motivation might have been masked by this effect.

Our finding that there was no significant increase in miss rates or mean RTs in the reinforced conditions, nor any trend in that direction, fails to support Hypothesis 2, namely that participants would tend to slow their Go responses globally when a greater incentive was provided for successfully inhibited Stop trials, regardless of the cost in missed Go trials. However, it may well be the case that such a strategy might be adopted under conditions in which the time limit for Go responses was less tight, or when the incentive to inhibit was increased simply by modification of the task instruction, as in the Band et al. study (2003), in which participants were either asked to respond as fast as possible, or to balance speed with accuracy, but in which no set time-limit was imposed.

The finding that the Go-Stop differences differed by motivational condition, and tended to be greater where the incentive to inhibit was greater, indicates support for Hypothesis 3: that participants would calibrate the degree of inhibition applied to the Go process in such a way as to optimize their overall probability of success on both trial types, and thus timing their responses to fall within the “sweet spot” indicated in Fig. 4. In the absence of a significant effect of condition on the skewness statistic, this effect alone would not differentiate between Hypothesis 2 and Hypothesis 3. However the lack of any indication of increased miss rate, or any significant increase in mean Go RT, as would be predicted under Hypothesis 2 means that the observed increase in Go-Stop RT difference can be regarded as support for Hypothesis 3. Thus, the behaviour of children under different motivational conditions was emulated by that of our simulated reinforcement learning model.

As expected, the children’s hit rate stabilized at a high level, and there was no significant main effect of motivational condition on overall Go RTs. This suggests that the children placed an intrinsic value on speedy responses, whether or not Go trials were extrinsically rewarded. This interpretation was supported by a subsequent trial-by-trial analysis of the children’s behavioural data, indicating that where there were two consecutive Go trials, the RT on the second trial tended to be shorter, *t*(31) = 6.42, *p* < .001, a finding also reported by Emeric et al. (2007). This is also indicated by the fact that the significantly greater Go-Stop RT difference where there was greater incentive to inhibit was reflected in an only slight increase in mean Go RTs that was coupled with an actual drop in failed Stop RTs. This suggests that, as with the model, the value placed on maintaining a high hit rate prevented a global shift of the distribution rightwards, and therefore resulted in a lower mean value in the lower half of the distribution, from which failed Stop RTs would have been drawn.

It is probably worth noting that our third hypothesis predicts not only a greater difference between Go and failed Stop RTs under the more motivated conditions, but also a more negative skew to the distribution of Go RTs. However, because the sampling error of the skew statistic is high, a very large number of trials would be required to deliver the statistical power required to detect anything less than a very large between-condition effect. In our modelling exercise the effect size of condition on the skewness statistic was less than a quarter of the size of the effect on the Go-Stop RT difference; we can conclude, therefore, that our study lacked the power to detect an effect of condition on the skewness statistic of the Go RT distribution. Indeed, in our sample, the F ratio for the between-condition effect on skew was well below 1 for all participants.

### 3.1 General discussion

The high level of motivation reflected in the children’s comments and in their performance makes the failure to find any evidence in the empirical data for motivational modulation of SSRTs noteworthy, and suggests that at least in typically developing children, SSRT may be a simple function of processing speed and ballistic processes, and irreducible beyond a certain level, despite incentives to do so. What did appear to be under volitional control was inhibitory modulation of the Go process. This is consistent with the findings of Band et al. (2003) that in a cued Go/No-Go task, participants who were asked to try to balance speed with accuracy tended to respond more slowly on all trials than those who were simply asked to prioritize speed, and suggests that top-down “restraint” processes may have inhibited the preparation of the Go response on all trials. Of note, the role of restraint processes across two types of action control tasks, the Go/No-Go task by Band et al. (2003) and the Stop Signal Task employed here, is consistent with the suggestion that these tasks tap common cognitive processes that support the control of action more in general (Aron et al., 2007).

In our task, unlike that of Band et al. (2003), slow responses were penalized in the reinforced conditions, making global slowing of go responses a penalized option. It was therefore of interest to see whether participants would be able to learn, not merely to delay their responses, but to calibrate their timing in order to maximize the overall chance of success, including timely responses on Go trials as well as successfully inhibited Stop trials (although of course the tracking algorithm ensured that this remained close to 50%). Our findings suggest that they were indeed able to do so, and raise the possibility that inhibitory processes do not simply race against activation process, but can be coordinated synergistically in order to target an optimal response time window, delaying the response until close to the “deadline”, as proposed by Ollman (1973). In turn, the evolution of this fine-tuned balance when highly motivated is consistent with models suggesting more broadly that the timely valuation of gains and losses plays a key role in learning to control action (Montague et al., 2006; Schultz et al., 1997).

**Fig. 9:**
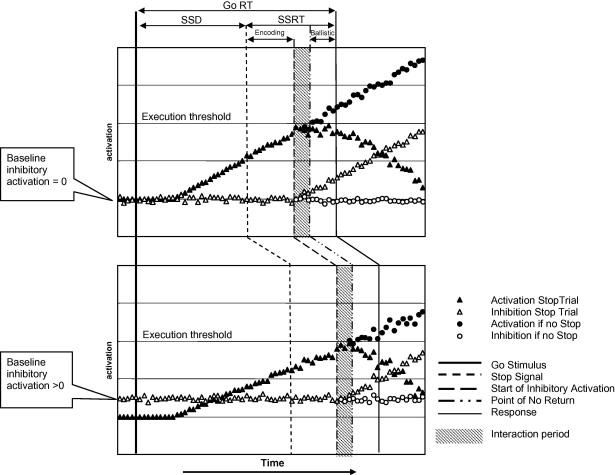
Proactive and reactive (phasic) inhibition. *Note*: The upper plot illustrates the model proposed by Boucher et al. (2007): in response to a Go stimulus, after a period of encoding, activation in neurons involved in preparation of the go response rise rapidly. The onset of the Stop signal is also followed by an encoding period, at the end of which activation rises rapidly in neurons that inhibit the rising go activation, rapidly arresting the rise. If the rise is arrested before an execution threshold is reached, the go response is successfully inhibited. The circles represent a Go trial, and the triangles a Stop trial in which the SSD is at a value that only just allows time for a successful inhibition. The shaded portion of the plot represents the time interval between the start of the rise in stopping process activation, and arrest of the rise in go process activation. The Go RT line represents the RT that would have occurred had the trial been a Go trial. The lower Figure illustrates our proposed model, in which proactive (baseline) pre-stimulus levels of activation in inhibitory circuits may be greater than zero, and also interact with the rising activation in the Go circuit, lengthening the RT should a response be made. Note that change in proactive level of activation in the inhibition circuits does not affect the observed SSRT.

In the context of Stop Signal Task performance specifically, and with regards to theories of action control more generally, one way of reconciling the evidence that the “horse race” between go and stopping processes are independent with evidence that inhibitory processes interact with the go processes might therefore be to extend the “interactive horse race” model of Boucher et al. (2007) to include an interaction between a proactive pre-stimulus restraint process and the go preparation response process. Boucher et al. (2007) suggest that activation in neurons responsible for inhibiting activation in go response preparation neurons rises rapidly following completion of the encoding of the Stop signal, and results, almost immediately, in the cancellation of the go response. This may be considered as a phasic *reactive* rise in activation of the inhibitory circuits consequent on presentation of the Stop signal. A schematic of their model is shown in the upper plot in Fig. 9. However, if we posit that go activation may also interact with pre-stimulus *proactive* activation levels in inhibitory circuits in the same manner, we find that the interaction has minimal impact on the measured SSRT, but has a major impact on the Go reaction time, as shown in the lower plot in Fig. 9. As well as providing a good fit to the behavioural data and computational model presented here, the effect of this additional parameter representing proactive inhibition to the model proposed by Boucher et al. (2007) is entirely consistent with the observed modulation of pre-stimulus event-related components such as the Lateralized Readiness Potential and the Contingent Negative Variation when participants are motivated to inhibit responses accurately, as reported by Band et al. (2003).

We suggest, therefore, that participants can voluntarily and proactively modulate the speed of the go process, possibly by active control of the level of pre-stimulus inhibition, and thus bringing the duration of their response-delay under volitional control. In our model runs, a slower “delay” was more likely to be selected for each trial when the “incentive” to inhibit was greater, resulting in both longer RTs for both failed Stop and Go trials. However, greater “incentive” to inhibit also resulted in a less positively (or more negatively) skewed distribution, increasing the difference between mean Go and mean failed Stop RTs. Our empirical data also showed this increase in RT difference, but superimposed on an overall trend for children to reduce their response time. Thus, while the participants increased their mean Go RT slightly when the motivation to succeed was greater as well, their mean failed Stop RTs, actually showed a decrease.

We propose that while the efficiency of the process by which a Stop signal is encoded may be subject to individual variation, resulting in between-subjects variability in observed SSRTs, the efficiency of the process is resistant to volitional control. Our findings suggest that the efficiency of the phasic, reactive, response to a countermanding stimulus has a ceiling that is readily achieved in typically developing children, and suggests that the horse-race model proposed by Logan and Cowan (1984) accounts largely for stimulus-driven aspects of inhibitory control.

Evidence that the phasic, reactive, response to a countermanding signal, as measured by the SSRT, tends to be of greater duration in disorders such as ADHD (Jennings et al., 1997; Logan et al., 1997; Nigg, 1999; Schachar et al., 1993) may therefore indicate slower speed-of-processing or inattention in such disorders, but does not in itself provide an explanation of impaired control of behaviour, such as that manifest as clinical levels of impulsivity, although clearly a child with longer reaction times or greater difficulty in attending to warning signals will require more time to comply with a countermanding instruction.

We note the fact that the RT difference between neutral and reinforced blocks was observed in data in which each motivational condition was presented in each of five blocks. This suggests that the effect was not dwarfed by practice effects, which in turn suggests that, while the children rapidly learned an effective strategy that they were willing to employ when motivated to succeed, the strategy remained effortful, and was therefore relaxed when the incentive to inhibit was lessened. One interpretation of this finding is that while these typically developing children were easily able to summon sufficient attentional resources to reach a plateau of efficiency with regard to their encoding of the stop stimulus, the capacity to restrain their impulse to respond was considerably more stretched.

The model and findings presented thus raise potentially interesting questions as to what processes might be deficient in conditions such as such as ADHD that are marked by apparently impulsive behaviour on tasks such as the Stop Signal Task. If the interactive model of Boucher et al. (2007) is correct, the consistent findings of lengthened SSRTs in children with ADHD (Jennings et al., 1997; Logan et al., 1997; Nigg, 1999; Schachar et al., 1993) would tend to implicate slow encoding and/or failure to initiate the stopping process on some trials (Band et al., 2003). However, consistent with the hypothesis made by Schachar et al. (2007), our model and findings suggest that proactive restraint processes may be more relevant to the clinical symptoms of impulsivity than slow encoding processes, and that the ability to delay the go response strategically may itself merit further investigation in children with ADHD. However, our model suggests that restraint is more likely to be indexed by the degree of RT slowing than by change in SSRTs.

Furthermore, as our findings suggest that typically developing children are not only able to delay their responses but to optimize their timing, it would be of interest to know whether this ability is reduced in children with ADHD. Impaired time production has been reported in children with ADHD (Bauermeister et al., 2005), giving reason to expect that children with ADHD might also show a reduced ability to learn to find the “sweet spot” required for optimal performance on tasks such as the Stop Signal Task. Smith and Brewer (1995) found that older adults were less able than young adults to target their responses within the “fast, safe” RT band, their RT adjustment being coarser than that of the younger group, and their RT variance thus greater. One possibility is that children and adolescents with ADHD may also struggle with fine RT adjustment, as would be supported by the robust finding of greater RT variability in Stop Signal Task data in these groups than in their age peers (Lijffijt et al., 2005). Further studies in which the learning model presented in this paper is lesioned in various ways in order to produce patterns of “behaviour” that might distinguish between causal deficits may shed light on these questions. Such attempts could also elucidate controversies on the effects of motivational incentives on Stop Signal Task performance in children with ADHD (Huang-Pollock et al., 2007; Konrad et al., 2000; Michel et al., 2005; Oosterlaan & Sergeant, 1998; Scheres et al., 2001; Slusarek et al., 2001). In conclusion, our current effort builds upon existing computational models as well as multiple behavioural and electrophysiological findings, but critically extends them by operationalising the parameters through which motivation modulates the control of action when the value assigned to the conflicting goals of speed and restraint are finely balanced.

## Figures and Tables

**Table 1:** Descriptive statistics: mean inhibition rates, miss rates, SSRTs, SSDs, Go RTs, failed Stop RTs, and Go-Stop RT differences by condition.

Measure	Neutral		Negative		*Positive*	
	*M*	*SD*	*M*	*SD*	*M*	*SD*
Inhibition rate	.49	.05	.49	.04	.49	.05
Miss rate	.13	.07	.12	.07	.13	.06
SSRT	219	44	223	46	222	45
SSD	229	75	237	72	237	77
Go RT	480	83	486	83	488	84
Stop (signal–respond) RT	446	74	444	72	437	66
Go RT – Stop RT	**34**	28	**42**	29	**51**	28

*Note:* Number of participants = 32. Rates are expressed as proportions and RTs are expressed in milliseconds. The only statistically significant difference in means (marked in bold) between conditions as tested by an one way ANOVA omnibus *F* test was for the Go-Signal Respond RT difference. A follow-up planned comparison indicated that the Go-Signal Respond difference in the neutral condition was significantly smaller than in the reinforced (“Positive” and “Negative”) conditions.

## References

[bib1] American Psychiatric Association (1994). *Diagnostic and statistical manual of mental disorders* (4th ed.). American Psychiatric Association.

[bib2] Aron A.R., Durston S., Eagle D.M., Logan G.D., Stinear C.M., Stuphorn V. (2007). Converging evidence for a fronto-basal-ganglia network for inhibitory control of action and cognition. Journal of Neuroscience.

[bib3] Baddeley A., DellaSala S. (1996). Working memory and executive control. Philosophical Transactions of the Royal Society B – Biological Sciences.

[bib4] Band G.P.H., Ridderinkhof K.R., van der Molen M.W. (2003). Speed-accuracy modulation in case of conflict: The roles of activation and inhibition. Psychological Research – Psychologische Forschung.

[bib5] Band G.P.H., van der Molen M.W., Logan G.D. (2003). Horse-race model simulations of the stop-signal procedure. Acta Psychologica.

[bib5a] Bauermeister J.J., Barkley R.A., Martinez J.V., Cumba E., Ramirez R.R., Reina G. (2005). Time estimation and performance on reproduction tasks in subtypes of children with attention deficit hyperactivity disorder. Journal of Clinical Child and Adolescent Psychology.

[bib6] Boucher L., Palmeri T.J., Logan G.D., Schall J.D. (2007). Inhibitory control in mind and brain: An interactive race model of countermanding Saccades. Psychological Review.

[bib7] Colonius H. (1990). A note on the stop-signal paradigm, or how to observe the unobservable. Psychological Review.

[bib8] De Jong R., Coles M.G.H., Logan G.D., Gratton G. (1990). In search of the point of no return – The control of response processes. Journal of Experimental Psychology – Human Perception and Performance.

[bib9] Duncan J. (2001). An adaptive coding model of neural function in prefrontal cortex. Nature Reviews Neuroscience.

[bib10] Emeric E.E., Brown J.W., Boucher L., Carpenter R.H.S., Hanes D.P., Harris R. (2007). Influence of history on saccade countermanding performance in humans and macaque monkeys. Vision Research.

[bib11] Goldman-Rakic P.S. (1996). The prefrontal landscape: Implications of functional architecture for understanding human mentation and the central executive. Philosophical Transactions of the Royal Society of London Series B – Biological Sciences.

[bib12] Huang-Pollock C.L., Mikami A.Y., Pfiffner L., McBurnett K. (2007). ADHD subtype differences in motivational responsivity but not inhibitory control: Evidence from a reward-based variation of the stop signal paradigm. Journal of Clinical Child and Adolescent Psychology.

[bib13] Jennings J.R., van der Molen M.W., Pelham W., Debski K.B., Hoza B. (1997). Inhibition in boys with attention deficit hyperactivity disorder as indexed by heart rate change. Developmental Psychology.

[bib14] Konrad K., Gauggel S., Manz A., Scholl M. (2000). Lack of inhibition: A motivational deficit in children with attention deficit/hyperactivity disorder and children with traumatic brain injury. Child Neuropsychology.

[bib15] Kornblum S., Hasbroucq T., Osman A. (1990). Dimensional overlap – Cognitive basis for stimulus–response compatibility – A model taxonomy. Psychological Review.

[bib16] Kramer A.F., Humphrey D.G., Larish J.F., Logan G.D., Strayer D.L. (1994). Aging and inhibition – Beyond a unitary view of inhibitory processing in attention. Psychology and Aging.

[bib17] Lappin J.S., Eriksen C.W. (1966). Use of a delayed signal to stop a visual reaction-time response. Journal of Experimental Psychology.

[bib18] Lijffijt M., Kenemans J.L., Verbaten M.N., van Engeland H. (2005). A meta-analytic review of stopping performance in attention-deficit/hyperactivity disorder: Deficient inhibitory motor control?. Journal of Abnormal Psychology.

[bib19] Logan G.D., Long J., Baddeley A.D. (1981). Attention, automaticity, and the ability to stop a speeded choice response. Attention and performance IX.

[bib20] Logan G.D. (1983). On the ability to inhibit simple thoughts and actions.1. Stop-signal studies of decision and memory. Journal of Experimental Psychology – Learning Memory and Cognition.

[bib20a] Logan G.D. (1992). Shapes of reaction-time distributions and shapes of learning-curves – a test of the instance theory of automaticity. Journal of Experimental Psychology-Learning Memory and Cognition.

[bib21] Logan G.D., Burkell J. (1986). Dependence and independence in responding to double stimulation – A comparison of stop, change, and dual-task paradigms. Journal of Experimental Psychology – Human Perception and Performance.

[bib22] Logan G.D., Cowan W.B. (1984). On the ability to inhibit thought and action – A theory of an act of control. Psychological Review.

[bib23] Logan G.D., Schachar R.J., Tannock R. (1997). Impulsivity and inhibitory control. Psychological Science.

[bib24] Michel J.A., Kerns K.A., Mateer C.A. (2005). The effect of reinforcement variables on inhibition in children with ADHD. Child Neuropsychology.

[bib25] Miller E.K., Cohen J.D. (2001). An integrative theory of prefrontal cortex function. Annual Review of Neuroscience.

[bib26] Montague P.R., King-Casas B., Cohen J.D. (2006). Imaging valuation models in human choice. Annual Review of Neuroscience.

[bib27] Nigg J.T. (1999). The ADHD response-inhibition deficit as measured by the stop task: Replication with DSM-IV combined type, extension, and qualification. Journal of Abnormal Child Psychology.

[bib28] Ollman R.R., Kornblum S. (1973). Simple reactions with random countermanding of the go signal. Attention and performance IV.

[bib29] Oosterlaan J., Sergeant J.A. (1998). Effects of reward and response cost on response inhibition in AD/HD, disruptive, anxious, and normal children. Journal of Abnormal Child Psychology.

[bib30] Osman A., Kornblum S., Meyer D.E. (1986). The point-of-no-return in choice reaction-time-controlled and ballistic stages of response preparation. Journal of Experimental Psychology – Human Perception and Performance.

[bib31] Passingham R.E. (1993). The frontal lobes and voluntary action.

[bib32] Ridderinkhof K.R., Band G.P.H., Logan G.D. (1999). A study of adaptive behavior: Effects of age and irrelevant information on the ability to inhibit one’s actions. Acta Psychologica.

[bib33] Schachar R.J., Logan G.D., Robaey P., Chen S., Ickowicz A., Barr C. (2007). Restraint and cancellation: Multiple inhibition deficits in attention deficit hyperactivity disorder. Journal of Abnormal Child Psychology.

[bib34] Schachar R.J., Tannock R., Logan G. (1993). Inhibitory control, impulsiveness, and attention-deficit hyperactivity disorder. Clinical Psychology Review.

[bib35] Scheres A., Oosterlaan J., Sergeant J.A. (2001). Response inhibition in children with DSM-IV subtypes of AD/HD and related disruptive disorders: The role of reward. Child Neuropsychology.

[bib36] Schultz W., Dayan P., Montague P.R. (1997). A neural substrate of prediction and reward. Science.

[bib37] Shallice T., Shallice T. (1988). From neuropsychology to mental structure. Neuropsychology to mental structure.

[bib38] Slusarek M., Velling S., Bunk D., Eggers C. (2001). Motivational effects on inhibitory control in children with ADHD. Journal of the American Academy of Child and Adolescent Psychiatry.

[bib39] Smith G.A., Brewer N. (1995). Slowness and age: Speed-accuracy mechanisms. Psychology and Aging.

[bib40] Stuss D.T., Alexander M.P. (2007). Is there a dysexecutive syndrome?. Philosophical Transactions of the Royal Society B – Biological Sciences.

[bib41] Sylwan R.P. (2004). The control of deliberate waiting strategies in a stop-signal task. Brazilian Journal of Medical and Biological Research.

[bib42] The MathWorks (2006). *MatLab*. Version 7.3.0.267 (R2006b).

[bib43] van den Wildenberg W.P.M., van der Molen M.W., Logan G.D. (2002). Reduced response readiness delays stop signal inhibition. Acta Psychologica.

[bib44] Wagenmakers E.J., Brown S. (2007). On the linear relation between the mean and the standard deviation of a response time distribution. Psychological Review.

